# Mst1 inhibits CMECs autophagy and participates in the development of diabetic coronary microvascular dysfunction

**DOI:** 10.1038/srep34199

**Published:** 2016-09-29

**Authors:** Jie Lin, Lei Zhang, Mingming Zhang, Jianqiang Hu, Tingting Wang, Yu Duan, Wanrong Man, Bin Wu, Jiaxu Feng, Lei Sun, Congye Li, Rongqing Zhang, Haichang Wang, Dongdong Sun

**Affiliations:** 1 Department of Cardiology, Xijing Hospital, Fourth Military Medical University, Xi’an, China; 2 Department of Cardiology, Tangdu Hospital, Fourth Military Medical University, Xi’an, China; 3 Department of Neurosurgery, Xijing Hospital, Fourth Military Medical University, Xi’an, China

## Abstract

Cardiovascular complications account for a substantial proportion of morbidity and mortality in diabetic patients. Abnormalities of cardiac microvascular endothelial cells (CMECs) lead to impaired cardiac microvascular vessel integrity and subsequent cardiac dysfunction, underlining the importance of coronary microvascular dysfunction. In this study, experimental diabetes models were constructed using Mst1 transgenic, Mst1 knockout and sirt1 knockout mice. Diabetic Mst1 transgenic mice exhibited impaired cardiac microvessel integrity and decreased cardiac function. Mst1 overexpression deceased CMECs autophagy as evidenced by decreased LC3 expression and enhanced protein aggregation when subjected to high glucose culture. Mst1 knockout improved cardiac microvessel integrity and enhanced cardiac functions in diabetic mice. Mst1 knockdown up-regulated autophagy as indicated by more typical autophagosomes and increased LC3 expression in CMECs subjected to high glucose cultures. Mst1 knockdown also promoted autophagic flux in the presence of bafilomycin A1. Mst1 overexpression increased CMECs apoptosis, whereas Mst1 knockout decreased CMECs apoptosis. Sirt1 knockout abolished the effects of Mst1 overexpression in cardiac microvascular injury and cardiac dysfunction. In conclusion, Mst1 knockout preserved cardiac microvessel integrity and improved cardiac functions in diabetic mice. Mst1 decreased sirt1 activity, inhibited autophagy and enhanced apoptosis in CMECs, thus participating in the pathogenesis of diabetic coronary microvascular dysfunction.

The prevalence of diabetes has reached 12.3% of the adult population in the USA with an increasing 1.7 million new diagnosed diabetic patients per year. The economic cost of diabetes and prediabetes was estimated to be US$322 billion in 2012^1^. Cardiovascular complications are major public health issues that account for a substantial proportion of morbidity and mortality in diabetic patients^2^. Our previous studies demonstrated that coronary microvascular dysfunction occurred in the early stages of diabetes as manifested by impaired microvascular barrier dysfunction, increased oxidative stress and apoptosis in CMECs^3^,^4^. However, little is known about the mechanisms underlying coronary microvascular damage associated with diabetes. Thus, to better manage diabetic patients and to prevent coronary microvascular damage, we sought to elucidate the pathogenesis of this dysfunction.

As the barrier between blood glucose and cardiomyocytes, the endothelium is believed to play a major role in the pathogenesis of diabetes-associated cardiovascular diseases (CVDs)^5^. Interestingly, autophagy has recently emerged as a potential novel target for the treatment of cardiovascular diseases. In particular, Lenoir and colleagues demonstrated that endothelial cell and podocyte autophagy synergistically protected patients from diabetes-induced glomerulosclerosis^6^. Nevertheless, no data exists on the role of CMECs autophagy in diabetes.

Mammalian ste20-like kinase 1 (Mst1) is a serine-threonine kinase that has been implicated in diverse biological functions, including autophagy, apoptosis and oxidative stress^7^,^8^,^9^. Mst1 has been reported to promote cardiac dysfunction in mice subjected to myocardial infarction (MI) through inhibition of autophagy^10^. Moreover, our previous study also demonstrated that up-regulating autophagy through Mst1 inhibition alleviates postinfarction cardiac dysfunction^11^. In the heart, Mst1 is widely distributed in cardiomyocytes and endothelial cells. Mst1 contains a ste20-related kinase catalytic domain in the amino-terminal segment followed by a regulatory domain at the COOH terminus^10^. Mst1 can directly inhibit the activity of silent information regulator 1 (sirt1)^12^. However, the direct role of Mst1/sirt1 signaling in the development of coronary microvascular damage in diabetes remains unknown. The objective of the present study was to investigate the precise involvement of autophagy and the underlying mechanisms in the pathogenesis of coronary microvascular disease in diabetes.

## Results

### Mst1 knockout preserves cardiac microvessel integrity and improves cardiac function in diabetic mice

**Figure 1:**
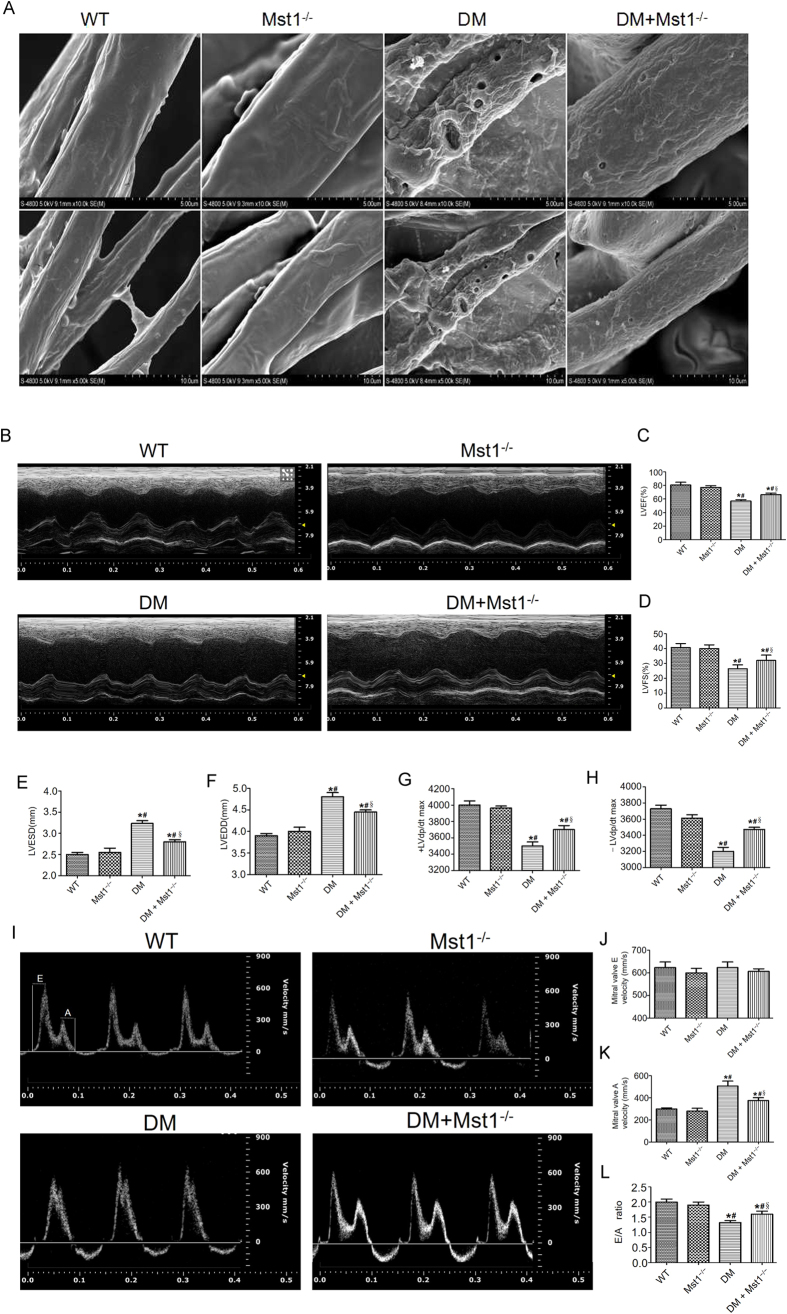
Mst1 knockout preserves cardiac microvessel integrity and improves cardiac function in diabetic mice. (**A**) Coronary microvascular structure evaluated by scanning electron microscopy (n = 5) (Magnification: upper panel x10,000; lower panel x5,000); (**B**) Representative M-mode echocardiograms (n = 6); (**C**) Representative mitral flow patterns from pulsed Doppler (n = 6); (**D,E**) LVEF and LVFS measurements; (**F,G**) LVESD and LVEDD measurements; (**H,I**) Hemodynamic evaluation of ±LV dp/dt max (n = 5); (**J**) Mitral valve E velocity; (**K**) Mitral valve A velocity; (**L**) Quantification of E/A ratio. LVEF, left ventricular ejection fraction; LVFS, left ventricular fraction shortening; LVESD, left ventricular end-systolic diameter; LVEDD, left ventricular end-diastolic diameter. The columns and error bars represent means and SE. *P < 0.05 vs WT; ^#^P < 0.05 vs Mst1^−/−^; ^§^P < 0.05 vs DM.

Coronary microvascular structure was evaluated by scanning electron microscopy. In non-diabetic mice, the surface of cardiac microvessels were smooth and well integrated. Cardiac microvascular integrity was significantly impaired in diabetic mice as evidenced by increased numbers of irregular exvaginations and invaginations. Interestingly, Mst1 knockout in diabetic mice preserved cardiac microvascular integrity (Fig. 1A).

To investigate whether Mst1 is involved in the development of cardiac dysfunction in diabetic mice, echocardiography and hemodynamic measurements were employed to evaluate the systolic and diastolic cardiac function. Diabetes led to impaired cardiac systolic function as manifested by decreases in left ventricular ejection fraction (LVEF) and left ventricular fraction shortening (LVFS), the effects of which were significantly alleviated by Mst1 knockout (Fig. 1B–D). Mst1 knockout inhibited left ventricular remodeling by decreasing left ventricular end-systolic dimension (LVESD) and left ventricular end-diastolic dimension (LVEDD) in diabetic mice (Fig. 1E,F). Consistently, Mst1 knockout improved the ±LV dp/dt max in diabetic mice (Fig. 1G,H). The E/A ratio, which is an indicator of cardiac diastolic function, was also enhanced by Mst1 knockout (Fig. 1I–L). The basic parameters of each group are shown in S-Table 1.

### Mst1 knockout enhances autophagy and reduces apoptosis in CMECs subjected to high glucose culture

**Figure 2:**
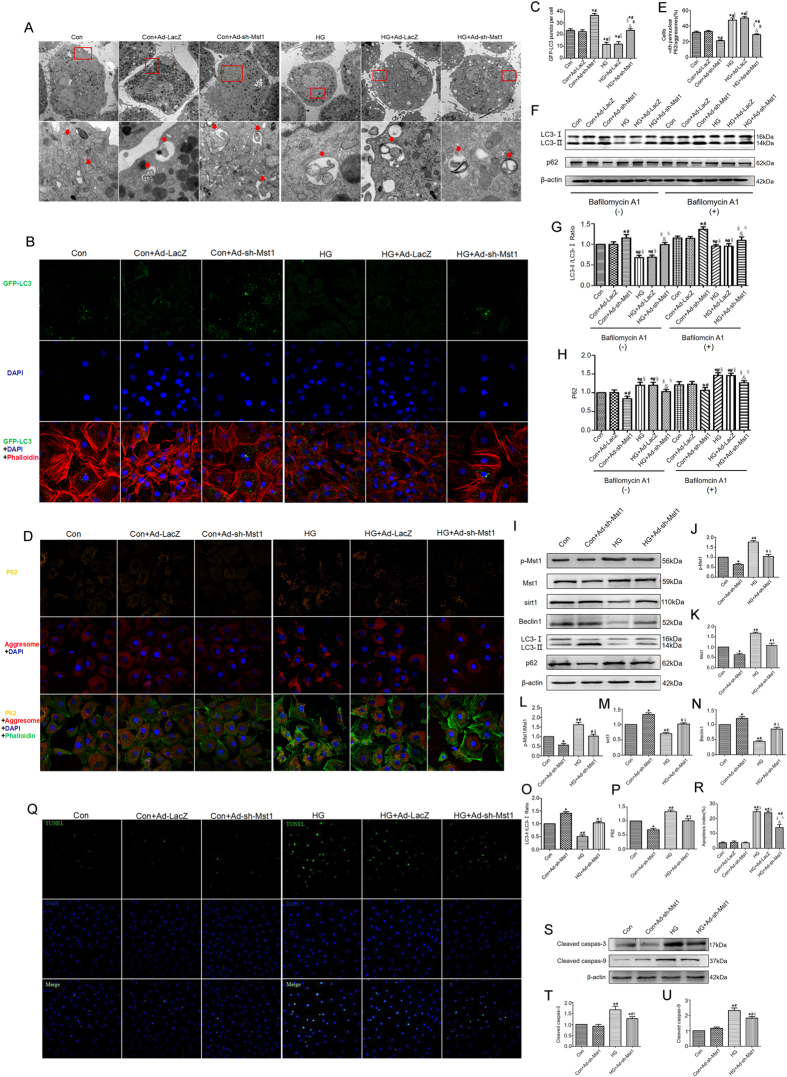
Mst1 knockout enhances autophagy while reduces apoptosis in CMECs subjected to high glucose culture. (**A**) Representative images of ultrastructural morphology and typical autophagosomes in CMECs subjected to different treatments. (Magnification: upper panel x6,000; arrow panel x26,500). Arrows in panels point to typical examples of autophagosomes covered by a double membrane; (**B,C**) Immunofluorescence staining and quantitative analysis of GFP-LC3 positive puncta. (**D**) Representative images of p62 (orange), DAPI (blue) and ProteoStat aggresome detection reagent (red) in cultured CMECs; (**E**) The number of p62 and aggresomes (p62/aggresomes) colocalized CMECs, indicated by yellow color; (**F–H**) Representative blots and analysis of LC3 and p62 in the absence or presence of bafilomycin A1. *P < 0.05 vs Con; ^#^P < 0.05 vs Con + Ad-LacZ; ^§^P < 0.05 vs Con + Ad-sh-Mst1; ^$^P < 0.05 vs HG; ^$^P < 0.05 vs HG + Ad-LacZ. L-P: Western blot and quantitative analyses of p-Mst1, Mst1, Sirt1, Beclin1, LC3-II/LC3-I and p62; (**Q,R**) Representative images of TUNEL staining and quantitative analyses of apoptotic cells; (**S–U**) Representative gel blots and quantitative analyses of cleaved caspase-3, cleaved caspase-9. The columns and error bars represent means and SE. *P < 0.05 vs Con; ^#^P < 0.05 vs Con + Ad-sh-Mst1; ^§^P < 0.05 vs HG.

As shown by transmission electron microscopy (TEM), autophagosome accumulation significantly increased in the Ad-sh-Mst1-transfected CMECs irrespective of culture on normal glucose or high glucose (Fig. 2A). An LC3-II positive puncta number, reflective of autophagosome abundance, was calculated for the CMECs transfected with GFP-LC3. Significant increases in the LC3-II puncta number in the Ad-sh-Mst1-transfected CMECs were determined for normal and high glucose conditions (Fig. 2B,C). We next determined whether Mst1 regulated protein and organelle quality control in CMECs by detecting the accumulation of aggresomes and p62. In CMECs cultured with high glucose medium, Ad-sh-Mst1 transfection significantly decreased the accumulation of aggresomes and p62 (Fig. 2D,E).

Consistently, Ad-sh-Mst1 transfection enhanced autophagic flux as evidenced by increased LC3-II/LC3-1 ratio and decreased p62 expression levels in the presence of bafilomycin A1, a lysosomal inhibitor used to evaluate autophagic flux (Fig. 2F–H). Additionally, elevated LC3-II/LC3-1 ratio and lower p62 expression levels were also demonstrated by Western blot in the Ad-sh-Mst1-transfected CMECs (Fig. 2I–P). The p-Mst1/Mst1 ratio increased, and Beclin1 expression decreased in the high glucose-cultured CMECs. This may have been the cause for the development of diabetic coronary microvascular dysfunction (Fig. 2I–N).

For apoptosis detection, fewer TUNEL-positive CMECs were observed in the Ad-sh-Mst1 transfection group when compared with the CMECs under high glucose conditions (Fig. 2Q,R). Expressions of cleaved caspase-3 and cleaved caspase-9 were also down-regulated in CMECs transfected with Ad-sh-Mst1 (Fig. 2S–U).

### Mst1 overexpression aggravates cardiac microvessel disorder and cardiac dysfunction in diabetic mice

**Figure 3:**
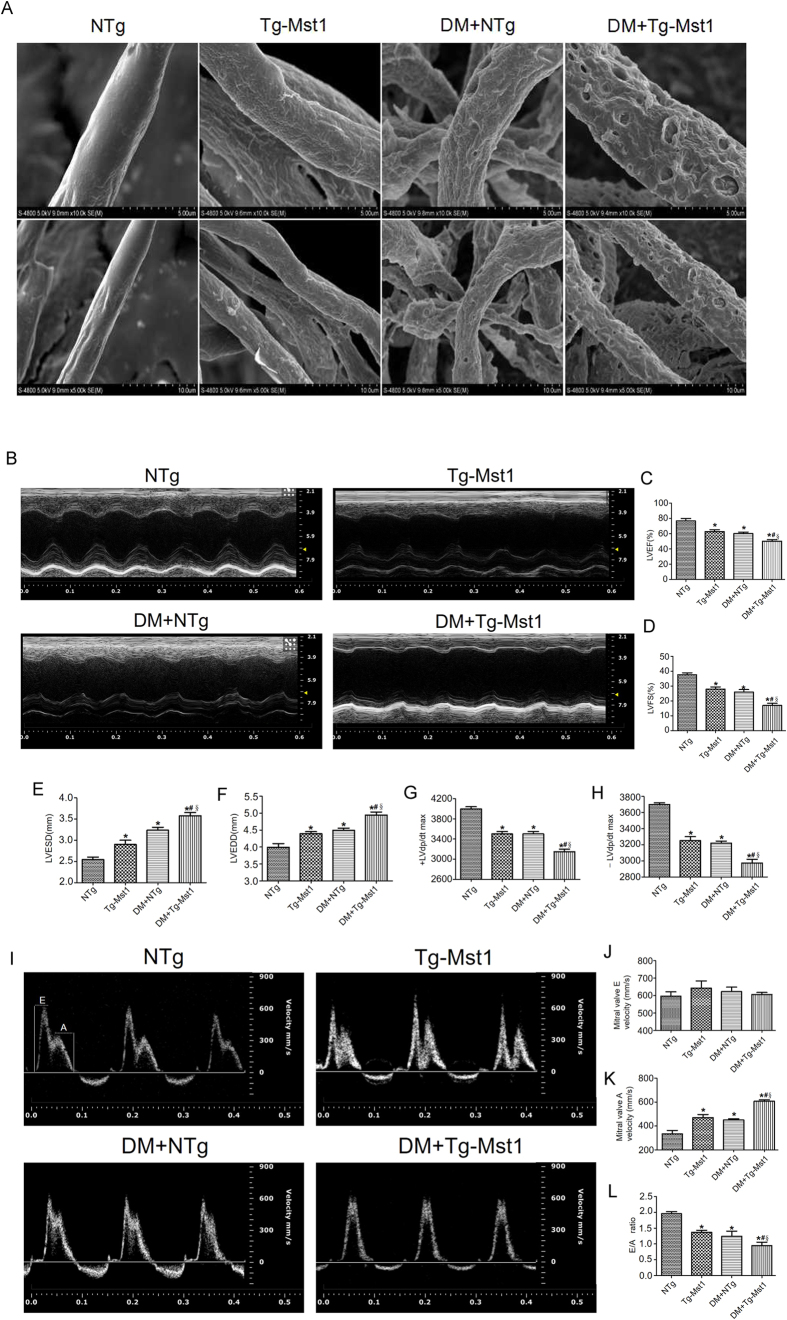
Mst1 overexpression aggravates cardiac microvessel disorder and cardiac dysfunction in diabetic mice. (**A**) Tg-Mst1 mice exhibited aggravated cardiac microvessel disorder evaluated by scanning electron microscopy (n = 5) (Magnification: upper panel x10,000; lower panel x5,000); (**B**) Representative M-mode echocardiograms (n = 6); (**C**) Representative mitral flow patterns from pulsed Doppler (n = 6); (**D,E**) LVEF and LVFS measurements; (**F,G**) LVESD and LVEDD measurements; (**H,I**) Hemodynamic evaluation of ±LV dp/dt max (n = 5); (**J**) Mitral valve E velocity; (**K**) Mitral valve A velocity; (**L**) Quantification of E/A ratio. LVEF, left ventricular ejection fraction; LVFS, left ventricular fraction shortening; LVESD, left ventricular end-systolic dimension; LVEDD, left ventricular end-diastolic dimension. The columns and error bars represent means and SE. *P < 0.05 vs NTg; ^#^P < 0.05 vs Tg-Mst1; ^§^P < 0.05 vs DM + NTg.

Diabetes disrupted cardiac microvascular integrity as manifested by the irregular exvaginations and invaginations in the surface of cardiac microvessels. Interestingly, as shown by scanning electron microscopy, cardiac microvascular integrity was further impaired in diabetic Mst1 transgenic (Tg-Mst1) mice (Fig. 3A). By measuring the LVEF and LVFS, echocardiographic analyses demonstrated that Mst1 overexpression aggravated cardiac systolic functions in diabetic mice. Diabetic Mst1 transgenic mice exhibited decreased LVEF and LVFS and increased LVESD and LVEDD when compared with the DM + NTg group (Fig. 3B–F). The E/A ratio is an index of cardiac diastolic function. Mst1 overexpression further decreased the E/A ratio in the DM + Tg-Mst1 group compared with the DM + NTg group (Fig. 3I–L). These data provide strong evidence that Mst1 may participate in the development of diabetic coronary microvascular dysfunction. The basic parameters in each group are shown in S-Table 2.

### Mst1 overexpression inhibits autophagy and increases apoptosis in CMECs subjected to high glucose culture

**Figure 4:**
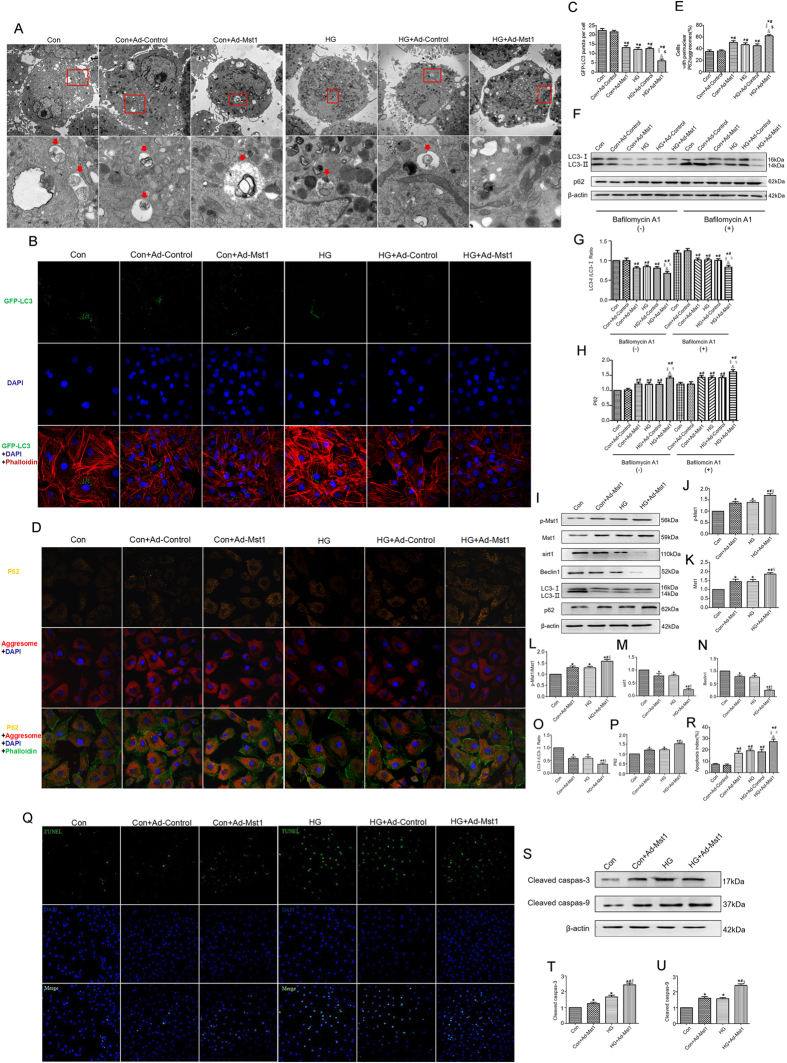
Mst1 overexpression inhibits autophagy while increases apoptosis in CMECs subjected to high glucose culture. (**A**) Representative images of ultrastructural morphology and typical autophagosomes in CMECs subjected to different treatments (Magnification: upper panel x6,000; arrow panel x26,500); (**B,C**) Mst1 overexpression decreased number of GFP-LC3 positive puncta; (**D,E**) Mst1 overexpression decreased the colocalization of p62 and aggresomes; (**F–H**) Representative blots and analysis of LC3 and p62 in the absence or presence of bafilomycin A1. *P < 0.05 vs Con; ^#^P < 0.05 vs Con + Ad-Control; ^§^P < 0.05 vs Con + Ad-Mst1. ^$^P < 0.05 vs HG; ^$^P < 0.05 vs HG + Ad-Control. (**L–P**) Western blot and quantitative analyses of p-Mst1, Mst1, sirt1, Beclin1, LC3-II/LC3-I and p62; (**Q,R**) Representative images of TUNEL staining and quantitative analyses of apoptotic CMECs; (**S–U**) Representative gel blots and quantitative analyses of cleaved caspase-3, cleaved caspase-9. The columns and error bars represent means and SE. *P < 0.05 vs Con; ^#^P < 0.05 vs Con + Ad-Mst1; ^§^P < 0.05 vs HG.

Ad-Mst1-transfected CMECs subjected to high glucose culture exhibited fewer autophagosomes when compared with the HG group (Fig. 4A). Furthermore, autophagy was also evaluated by GFP-LC3 transfection and aggresomes/p62 double staining. Ad-Mst1 transfection significantly decreased the number of LC3-positive puncta compared with the control group under normal glucose and high glucose conditions (Fig. 4B,C). Consistently, the accumulation of aggresomes and p62 was aggravated in the Ad-Mst1 group compared with the control group under normal glucose and high glucose conditions (Fig. 4D,E).

Ad-Mst1 transfection inhibited autophagic flux, as shown by a decreased LC3-II/LC3-Ι ratio and increased p62 expression levels after blocking lysosomal activity with bafilomycin A (Fig. 4F–H). Furthermore, Western blot analyses revealed a reduced LC3-II/LC3-Ι ratio and an accumulation of p62 in the CMECs transfected with Ad-Mst1 in normal glucose and high glucose cultures (Fig. 4I–P). Ad-Mst1 transfection resulted in significantly increased TUNEL-positive CMECs and cleaved caspase-3 and cleaved caspase-9 expression levels compared with the control group (Fig. 4Q–U). Overall, these data show that Mst1 overexpression inhibited autophagy and increased apoptosis in CMECs under high glucose culture.

### Mst1/sirt1 signaling is involved in the development of coronary microvascular damage in diabetes

**Figure 5:**
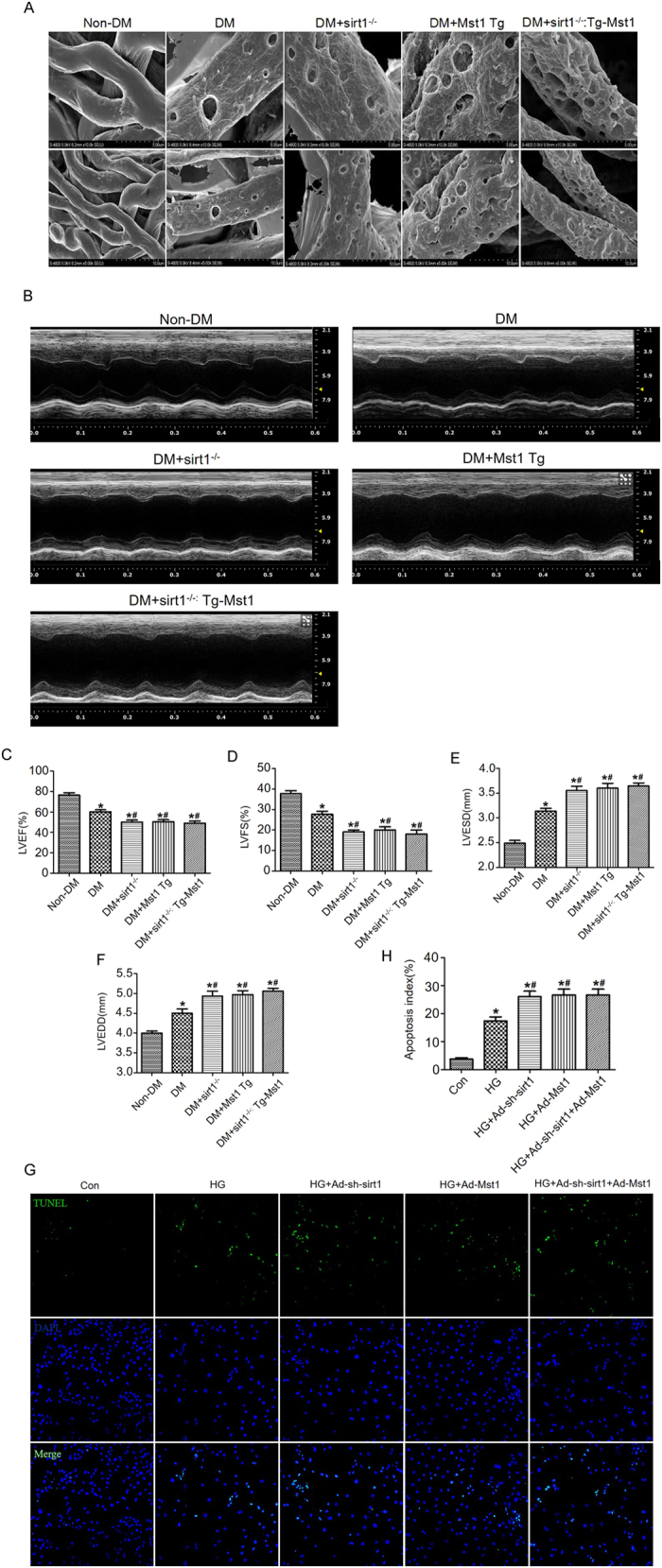
Mst1/sirt1 signaling is involved in the development of coronary microvascular damage in diabetes. (**A**) Cardiac microvessel integrity evaluated by scanning electron microscopy (n = 5); (**B–D**) Echocardiographic evaluation of LVEF and LVFS (n = 5) (Magnification: upper panel x10,000; lower panel x5,000); (**E,F**) LVESD and LVEDD measurements. *P < 0.05 vs non-DM; ^#^P < 0.05 vs DM. (**G,H**) Representative images of TUNEL staining and quantitative analyses of apoptotic CMECs. The columns and error bars represent means and SE. *P < 0.05 vs Con; ^#^P < 0.05 vs HG.

We crossed sirt1^−/−^ mice with Tg-Mst1 mice, to generate sirt1^−/−^: Tg-Mst1 mice and constructed a diabetic model. Diabetic sirt1^−/−^ mice and Tg-Mst1 mice exhibited impaired cardiac microvascular integrity compared with diabetic mice (Fig. 5A). Diabetic sirt1^−/−^: Tg-Mst1 mice demonstrated similar levels of cardiac microvascular integrity as diabetic sirt1^−/−^ mice, which indicated that the sirt1 knockout abolished the effects of Mst1 overexpression on cardiac microvascular injury (Fig. 5A).

As shown by echocardiography, diabetic sirt1^−/−^: Tg-Mst1 mice exhibited similar decreases in LVEF and LVFS as in diabetic sirt1^−/−^ mice (Fig. 5B–D). *In vitro* studies also demonstrated that Mst1 overexpression did not further increase the apoptotic index of high glucose-cultured CMECs when compared with the HG + Ad-sh-sirt1 group (Fig. 5G,H). These data indicated that Mst1 overexpression likely induced coronary microvascular damage through sirt1 inhibition.

## Discussion

The prevalence of type 1 diabetes is increasing worldwide at a rate of 3–5% annually^13^. However, despite the development of improved strategies for glucose control, the risk of vascular complications remains unacceptably high in diabetic patients. Coronary microvascular disease, an early change in diabetic cardiomyopathy, is one of the determinants of left ventricular dysfunction^14^. The present study revealed impaired left ventricular diastolic and systolic function in diabetic mice. Cardiac microvascular integrity was also compromised in diabetes. We demonstrated, for the first time, the importance of CMECs autophagy in the maintenance of coronary microvascular functions under diabetic conditions.

In diabetic patients, autophagy may serve as a coping mechanism in response to diabetic stress. Accumulating evidence and our previous study demonstrated that diabetes substantially impaired the properties of the endothelium and resulted in endothelial dysfunction, which could lead to the development of cardiovascular diseases^15^,^16^. Autophagy is the targeted degradation of proteins and organelles critical for homeostasis and endothelial cell survival^17^. Autophagy has been demonstrated to be a protective mechanism in human CMECs subjected to I/R injury^18^. However, changes in autophagy remain unclear in CMECs when CMECs are subjected to diabetic stress. Thus, we investigated the importance of CMECs autophagy in the context of diabetes. In the present study, decreased LC3 expression and enhanced protein aggregation were observed in diabetic hearts as evidenced by immunofluorescence and Western blot analysis. By using the lysosomal inhibitor bafilomycin A1, autophagic flux was observed to be inhibited in diabetic hearts.

Interestingly, we demonstrated that diabetic cardiomyopathy (DCM) is associated with the suppression of cardiac autophagy and the enhancement of cardiomyocyte apoptosis. The ability of Mst1 to regulate both autophagy and apoptosis may contribute to the progression of DCM. Nevertheless, little is known about the mechanism mediating CMECs autophagy. This prompted the investigation to determine whether Mst1 contributed to CMECs dysfunction in diabetes. As expected, CMECs subjected to high glucose culture exhibited increased Mst1 phosphorylation. Mst1 knockout significantly alleviated diabetes-induced cardiac dysfunction and microvascular injury without affecting non-diabetic hearts. These observations provide strong evidence in support of the hypothesis that CMECs autophagy inhibition by Mst1 is a critical event in the development of coronary microvascular injury in the diabetic heart.

The mammalian sirtuin family consists of seven members, sirt1-7. Sirt1 is the most widely studied sirtuin homologue and has been shown to play an important role in cellular energy metabolism^19^,^20^. Sirt1 has also been implicated in glucose homeostasis and lipid metabolism in various tissues and may serve as a potential pharmacological target for fighting metabolic and vascular diseases^21^. Yuan and colleagues reported that sirt1 activity can be inhibited by Mst1. When Mst1 transgenic mice were subjected to diabetic injury, sirt1 expression decreased. However, Mst1 knockout diabetic mice exhibited increased sirt1 expression levels. Intramyocardial Mst1 adenovirus injection did not aggravate cardiac dysfunction when sirt1 knockout mice were subjected to diabetic injury. Diabetic sirt1^−/−^: Tg-Mst1 mice exhibited similar cardiac microvascular integrity and cardiac function as the diabetic sirt1^−/−^ mice. These results suggested that sirt1 functions downstream of Mst1 in the pathogenesis of coronary microvascular injury in the diabetic heart.

## Conclusions

Collectively, our data demonstrate that autophagy inhibition in CMECs plays an important role in coronary microvascular dysfunction in diabetes. Sirt1 may function downstream of Mst1 in the development of diabetic coronary microvascular dysfunction.

## Materials and Methods

### Animals and Procedures

Mst1 knockout (Mst1^−/−^), Mst1 transgenic (Tg-Mst1) and sirt1 knockout (sirt1^−/−^) mice were purchased from K&D gene technology (WuHan, China) (C57BL/6 background). The sirt1^−/−^ mice were crossed with Tg-Mst1 mice to generate sirt1^−/−^: Tg-Mst1 mice. Western blot and real-time PCR (RT-PCR) were used to screen Mst1^−/−^, Tg-Mst1, sirt1^−/−^ and sirt1^−/−^: Tg-Mst1 mice (2 months of age). Mice received humane care in adherence with the National Institutes of Health Guidelines on the Use of Laboratory Animals. Protocols were approved by the Fourth Military Medical University Committee on Animal Care.

Diabetes were induced by intraperitoneal (i.p.) injections of streptozocin (STZ, 50 mg/kg; STZ was dissolved in 0.1 M citrate buffer, pH 4.5) for 5 days and maintained for another 3 months as previously described^22^. Animals with glucose levels no less than 16.6 mmol/L were classified as diabetes (DM). Mice were divided into the following groups: (i) Wide-type (WT); (ii) Mst1^−/−^; (iii) DM; (iv) DM + Mst1^−/−^; (A) Non-transgenic mice (NTg); (B) Tg-Mst1; (C) DM + NTg; (D) DM + Tg-Mst1; ① Non-DM; ② DM; ③ DM + sirt1^−/−^; ④ DM + Mst1 Tg; ⑤ DM + sirt1^−/−^: Tg-Mst1.

### Cardiac microvessel integrity examination

Scanning electron microscopy was used to evaluate microvascular endothelial integrity as previously described^3^. Mice hearts were perfused with 5 mL low viscosity resin mixed with benzoyl peroxide through the aorta and were dissolved in a 5% sodium hydroxide solution at room temperature. After 3–4 days, the connective tissue surrounding the blood vessels were removed. The isolated cardiac microvessels were prepared by standard methods, including dehydration, desiccation, and gilding before observed by a scanning electron microscope (Hitachi S-3400N). The magnification was 10,000 in the upper panel and 5,000 in the lower panel.

### Cardiac function measurement

M-mode echocardiography was conducted using an echocardiography system with a 15-MHz linear transducer (VisualSonics Vevo 2100, Toronto, Canada) as previously described^22^. Mice were anesthetized with 1.5–2.0% isoflurane. LVEF, LVFS, LVESD, LVEDD and the E/A ratio were calculated by computer algorithms. The first derivative of the left ventricular pressure (+dP/dt max and −dP/dt max) was measured via a Millar Mikro-tip catheter transducer that was inserted into the left ventricular cavity through the left carotid artery. The LV ± dP/dt were obtained by computer algorithms and an interactive graphics program (Po-Ne-Mah Physiology Platform P3 Plus, Gould Instrument Systems, Valley View, Ohio)^23^.

### Isolation, cultivation and identification of CMECs

CMECs were isolated as previously described with minor modifications^3^. Briefly, left ventricles were excised from male non-transgenic mice (6 weeks, 20–30 g) and rinsed with PBS supplemented with heparin. The left ventricle was removed, cut into pieces and incubated in 0.2% collagenase type II (Sigma, St, Louis, Mo) solution for 5–7 min until the tissue blocks disappeared, followed by 0.25% trypsin (Sigma) for another 3–5 min at 37 °C in a water-bath heater. Cells were centrifuged and re-suspended in complete medium containing 20% fetal bovine serum (Hyclone, USA) and maintained at 37 °C in the presence of 20% O_2_, 5% CO_2_ and 75% N_2_. A dil-acetylated low-density lipoprotein (Molecular Probes, Eugene, OR) intake assay was used to identify the CMECs. The purity of the CMECs was 90.4 ± 2.1%. Passage 2–3 cells were used for further studies. The glucose concentrations in the normal glucose and high glucose media were 5.5 mmol/L and 33 mmol/L, respectively.

### Adenoviruses construction and transfection

The adenoviruses harboring GFP-LC3 (GFP-LC3) (MOI: 100) was purchased from GeneChem technology Ltd (Shanghai, China). The adenoviruses harboring Mst1 (Ad-Mst1) (MOI: 100), Mst1 shRNA (Ad-sh-Mst1) (MOI: 100), control vectors for Ad-Mst1 (Ad-Control) and Mst1 shRNA (Ad-LacZ) were purchased from Hanbio Technology, Ltd (Shanghai, China) and were transduced 24 h after transduction of GFP-LC3 as previously described^11^. The shRNA sequence targeting mouse Mst1 was 5′-GCCCTCACGTAGTCAAGTATT-3′. After 36 h, the CMECs were treated with low (5.5 mM) or high (33 mM) glucose for 48 h.

### Fluorescence detection of GFP-LC3, aggresomes and p62

Fluorescence detection of GFP-LC3, aggresomes and p62 were conducted according to manufacturer instructions as previously described^11^.

### CMECs apoptosis determination

CMECs apoptosis was detected by terminal deoxyribonucleotidyl transferase-mediated dUTP-biotin nick end labeling (TUNEL) assay using a Cell Death Detection Kit (Roche, Penzberg, Germany) according to manufacturer instructions. The apoptosis index was expressed as a percentage of the number of TUNEL-positive CMECs to the total number of CMECs^23^. The expression levels of cleaved caspase-3 and cleaved caspase-9 were detected by Western blot.

### Western blot analysis

Western blot was performed as previously described^11^. The following antibodies were used: Mst1, p-Mst1, sirt1, Beclin1, and p62 (Abcam, Cambridge, MA, UK); LC3A/B (Cell Signaling, Danvers, MA, USA); cleaved caspase-3 and cleaved caspase-9 (Sigma, St. Louis, MO, USA); and β-actin (Santa Cruz, CA, USA). The signals were quantified by Image ProPlus software (Media Cybernetics, MD Rockville, USA).

### Statistics

Continuous variables that approximated the normal distribution were expressed as means ± SE. Comparison between groups were subjected to ANOVA followed by Bonferroni correction for post host test. Two sided tests have been used throughout, and P values < 0.05 were considered statistically significant. SPSS software package version 17.0 (SPSS, Chicago, IL) was used for data analysis.

## Additional Information

**How to cite this article**: Lin, J. *et al*. Mst1 inhibits CMECs autophagy and participates in the development of diabetic coronary microvascular dysfunction. *Sci. Rep*. **6**, 34199; doi: 10.1038/srep34199 (2016).

## Supplementary Material

Supplementary Information
