# Supplementary material for: Mst1 inhibits CMECs autophagy and participates in the development of diabetic coronary microvascular dysfunction
Source: Sci Rep. 2016 Sep 29;6:34199. doi: 10.1038/srep34199 (PMC5040958; doi:10.1038/srep34199)
Supplement: Supplementary Information [file srep34199-s1.doc]

**Title:**

Mst1 inhibits CMECs autophagy and participates in the development of diabetic coronary microvascular dysfunction

**Authors:**

Jie Lin^1,3#^, M.D., Lei Zhang^2#^, M.D., Ph.D., Mingming Zhang^1,3#^, M.D., Jianqiang Hu^1,3#^, M.D., Tingting Wang^1^, M.D., Yu Duan^1^, M.D., Wanrong Man^1^, M.D., Bin Wu^1^, M.D., Jiaxu Feng^1^, M.D., Lei Sun^1^, M.D., Congye Li^1^, M.D., Rongqing Zhang^1^, M.D., Haichang Wang^3^*, M.D., Ph.D., Dongdong Sun^1,3^*, M.D., Ph.D

^1^Department of Cardiology, Xijing Hospital, Fourth Military Medical University, Xi’an, China;

^2^Department of Neurosurgery, Xijing Hospital, Fourth Military Medical University, Xi’an, China;

^3^Department of Cardiology, Tangdu Hospital, Fourth Military Medical University, Xi’an, China;

^#^ Contributed equally to this work.

*Correspondence: Dongdong Sun, 127 West Changle Road, Department of Cardiology, Xijing Hospital, Fourth Military Medical University, Xi’an, Shaanxi, 710032, China. Fax: 86 29 84775183; Tel: 86 29 84775183;

E-mail: [wintersun3@gmail.com](mailto:wintersun3@gmail.com) (DS).

Haichang Wang, 1 Xinsi Road, Department of Cardiology, Tangdu Hospital, Fourth Military Medical University, Xi’an, Shaanxi, 710032, China.

Fax: 86 29 84773469; Tel: 86 29 84773469; E-mail: [wanghc@fmmu.edu.cn](mailto:wanghc@fmmu.edu.cn) (HW).

**Supplementary Materials**

S-Table 1. Basic parameters of mice

| Parameter | WT | Mst1^−/−^ | DM | DM+ Mst1^−/−^ |
| --- | --- | --- | --- | --- |
| Body weight (g)  Heart rate (bpm) | 32(1)  471(20) | 33(1)  470(21) | 25(1) *  463(26) | 29(2) ^#^  465(24) |

Data were expressed as Mean (SEM), n = 12 to 13 mice per group, *p<0.05 vs. WT group, #p <0.05 vs. DM group.

S-Table 2. Basic parameters of mice

| Parameter | NTg | Tg-Mst1 | DM+ NTg | DM+ Tg-Mst1 |
| --- | --- | --- | --- | --- |
| Body weight (g)  Heart rate (bpm) | 31(1)  470(20) | 30(2)  464(25) | 25(1) *  465(23) | 21(2) *^#^  469(21) |

Data are expressed as Mean (SEM), n = 12 to 13 mice per group, *p<0.05 vs. NTg group, #p <0.05 vs. DM+ NTg group.
